# Benign paediatric liver tumours: The radiological maze demystified

**DOI:** 10.4102/sajr.v28i1.2726

**Published:** 2024-02-12

**Authors:** Poonam Sherwani, Devasenathipathy Kandasamy, Raju Sharma, Prabudh Goel, Manisha Jana, Nellai Krishnan

**Affiliations:** 1 Department of Radiodiagnosis, All India Institute of Medical Sciences, Rishikesh, India; 2 Department of Radiodiagnosis, All India Institute of Medical Sciences, Delhi, India; 3 Department of Paediatric Surgery, All India Institute of Medical Sciences, Delhi, India

**Keywords:** benign, liver, infantile hepatic haemangioma, mesenchymal hamartoma, adenoma, focal nodular hyperplasia

## Abstract

The trajectory from the clinical identification of a benign liver mass to establishing a precise diagnosis is perplexing and arduous. Related contributory factors are that such lesions are encountered infrequently and that there is a nonavailability of dedicated paediatric radiologists in the developing world. The objective of this study was to review the spectrum of benign liver lesions in children and their typical imaging features. Cross-sectional imaging of all paediatric patients (< 18 years) with liver lesions (single and multiple) performed in the institute from 01 January 2018 to 01 January 2019 as well as those acquired at outside institutions and referred to the institute for management was included. Ultrasound was done as the first line of investigation in all the cases with suspicious liver masses and retrospectively performed in referral cases in whom CT or MRI was already done. Images were analysed by two senior radiologists. Most of the cases were diagnosed based on clinical, biochemical and imaging findings, and biopsy was only performed in equivocal cases. Most of the benign liver lesions in the paediatric age group were hepatic haemangioma and mesenchymal hamartomas. A simplified clinical-radiologic paradigm should be established for benign liver lesions in children to assist in reaching the correct diagnosis.

**Contribution:**

The article demonstrates the salient radiological findings of various benign liver lesions in the paediatric age group and the role of demographic, clinical and biochemical findings, which plays a substantial role in the diagnosis and avoids unnecessary biopsies.

## Introduction

Knowledge of common benign paediatric liver masses and their imaging appearances is essential for radiologists to suggest a scientifically appropriate list of differential diagnoses. While evaluating liver tumours in children, apart from the imaging appearance of the lesions, it is important to consider the age of a child, clinical features and biochemical markers to assist in reaching the correct diagnosis. It is also important to be aware of the appropriate modality to be used for the diagnosis, as radiation exposure and motion artefact are two major concerns in paediatric imaging that can influence the choice of the imaging modality. Therefore, ultrasound is the initial investigation of choice to diagnose and follow-up these children. Cross-sectional modalities like CT and MRI are needed for further characterisation, to look for extent and metastases.

This review addresses the classification of liver tumours and the typical imaging appearance of common benign hepatic tumours in infants and children. Besides the clinical presentation, age incidence and various laboratory markers which can provide important clues to arrive at a list of differential diagnosis are also discussed.

### Classification of paediatric liver tumours

**FIGURE 1:**
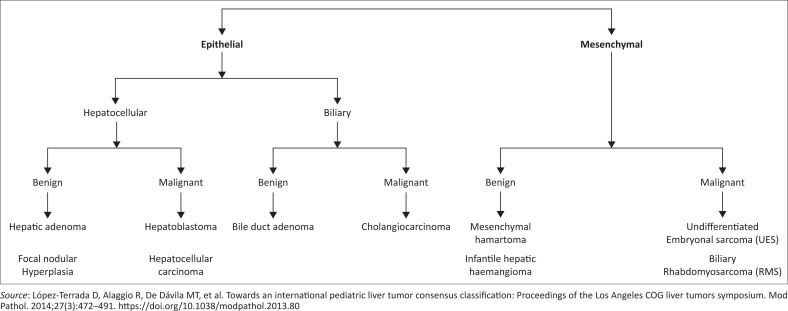
Classification of paediatric liver tumours.

The latest International Paediatric Liver Tumour classification (2014) has been summarised in Figure 1.^1^

### Epidemiology and clinical features

Clinical profile and epidimology has been described in Table 1.

**TABLE 1:** Epidemiology and clinical profile of benign liver tumours.

Tumour	Age group	Associated clinical features
Hepatic haemangioma	< 1 year	Hypothyroidism
		Cardiac failure
		Coagulopathy
		Cutaneous haemangioma
		AFP: normal
Mesenchymal hamartoma	< 5 years	Foetal hydrops
		Neonatal respiratory distress
		Cardiac failure
		AFP: normal
Focal nodular hyperplasia	Young children, adolescents	Usually asymptomatic
	Strong predilection in girls	Detected incidentally
		AFP: normal
Hepatic adenoma	> 10 years	Steroid use
		Glycogen storage disease
Nodular regenerative hyperplasia	Very rare. Youngest age – adolescence	Various systemic diseases like myeloproliferative syndrome, thrombocytopaenia, pancytopaenia, collagen vascular disease and Budd-Chiari syndrome

AFP, alpha-foetoprotein protein; USG, ultrasound; NCCT, non-contrast CT scan; EOB-DTBA, gadolinium ethoxybenzyl diethylenetriamine pentaacetic acid; HCC, hepatocellular carcinoma.

### Imaging modalities

#### Ultrasonography

Ultrasound is the primary investigation of choice for the evaluation of liver masses in children because of its easy availability, real-time evaluation capability, low cost, non-requirement for sedation and lack of ionising radiation. Apart from confirming the presence of the mass and number of lesions, ultrasound also depicts the solid or cystic nature of the mass which helps in narrowing the differential diagnosis. With the help of colour Doppler, the vascularity of the lesion can be assessed. Vascular structures like the hepatic artery, portal vein and hepatic vein can be evaluated in relation to the lesion.^2^ Besides the diagnosis, ultrasound can also help guide interventions such as tissue sampling which can be performed successfully with very low complication rates.

Contrast-enhanced ultrasound (CEUS) is a valuable addition that can be used to characterise lesions better and study their contrast kinetics, similar to contrast-enhanced CT (CECT) and contrast-enhanced MRI (CEMRI). Ultrasound contrast media can be safely used even in patients where CT and MRI contrast agents are contraindicated. Ultrasound contrast agents are neither nephrotoxic nor hepatotoxic and can be administered in patients with renal failure. With CEUS, one can evaluate the enhancement characteristics of a lesion in real time, unlike CT and MRI which are usually snapshot techniques. However, CEUS has a few disadvantages such as subjectivity and difficulty in evaluating multiple lesions.^3^ In comparison to conventional ultrasound, CEUS is superior in the diagnosis of various focal liver lesions. Contrast-enhanced ultrasound has well-documented safety data in the adult population and in children with hepatic lesions and vesicoureteric reflux.^4^

#### Computed tomography

Despite the risk of radiation exposure, CT is often used to further characterise focal liver lesions and to evaluate the extent of involvement as part of the preoperative assessment. To minimise the radiation exposure, non-contrast CT (NCCT) can be avoided, as calcification can be easily detected on contrast-enhanced scans as well. Although multiphase scanning is performed routinely for liver lesions in adults, in an attempt to lower the radiation dose, the same is not recommended in children. Single portal venous phase scans are usually sufficient to make the diagnosis in children. Multiphase scans are reserved only in situations where MRI is either not available or feasible. When a multiphase scan is performed, the arterial phase is acquired by scanning 15 s – 20 s after the start of contrast injection. Portal venous phase is acquired at 60 s – 90 s and delayed phase or equilibrium phase at 3 min – 5 min. Nonionic contrast is injected at a rate of 1.2 mL/s – 2 mL/s depending on the size of the intravenous cannula. The dose administered should be 2 mL/kg or a maximum of 150 mL. Sedation or anaesthesia is necessary only in infants and small children.^4^

#### Magnetic resonance imaging

MRI is usually the best modality to evaluate liver lesions and its utility is on the rise because of wider availability. The inherent contrast resolution is much better than CT and there are multiple paradigms and parameters which can be evaluated on MRI. It can characterise the lesion without any radiation, and is preferred over CT in children in whom iodinated contrast agents are contraindicated. It also has the advantage of evaluating the lesion in the hepatobiliary phase when using hepatobiliary-specific contrasts, especially for diagnosing focal nodular hyperplasia (FNH).^4^

The characteristic features of different benign liver masses in children on ultrasound, CT and MRI have been summarised in Table 2^4,5^.

**TABLE 2:** Summary of imaging characteristics in benign liver tumours.

Diagnosis	Antenatal diagnosis (Y/N)	Antenatal USG	Antenatal MRI	USG	USG Doppler	CEUSG	CT	MRI
Hepatic haemangiomas	Y	Mixed solid and cystic hepatic mass with increased blood flow on colour Doppler	T1W: hypointense T2W: hyperintense	Hypoechoic lesion. Occasionally calcification.	Increased vascularity	Arterial phase: peripheral nodular discontinuous enhancement Portal venous phase: gradual centripetal fill-in	Contrast enhancement in the periphery with little contrast in the centre. Occasional calcification	T1W: hypointense T2W: hyperintense
Mesenchymal hamartoma	Y	Either solid or complex, multicystic, well-circumscribed lesion	T1W: hypointense T2W: hyperintense	Predominantly cystic or mixed, cystic-solid	Minimal vascularity	–	Multicystic or solid-cystic. Septae and solid portion show enhancement	T1W: hypointense T2W: variable signal. Septa and wall hypointense on both. Mild enhancement post contrast.
Focal nodular hyperplasia	N	–	–	Well-circumscribed mass with variable echogenicity and a central hyperechoic scar	Spoke wheel type of vascularity with increased flow in the central scar	Arterial phase: centrifugal hyperenhancement Portal venous phase: isoenhancing, no washout	Hypo- to isodense on NCCT and homogeneous enhancement post contrast. Central scar shows delayed enhancement. No calcification	T1W: hypo- or isointense. Scar hypointense T2W: hyper- to isointense. Scar hyperintense and shows delayed enhancement
Hepatic adenoma	N	–	–	Heterogeneous with areas of hyperechogenicity due to fat and haemorrhage	Intratumoural vessels associated with either pulsatile or continuous peripheral flow	Arterial phase: hypervascular relative to the adjacent liver Portal venous phase: isoechoic, shows partial washout	Heterogeneous: presence of fat, calcification, haemorrhage. No central scar. Enhancement in the arterial phase, washout in the delayed phase	T1W: heterogeneous T2W: heterogeneous Fat hyperintense on both T1W and T2W
Nodular regenerative hyperplasia	N	–	–	Multiple iso- to hypoechoic well-circumscribed nodules	Vascularity within some of these lesions	–	Hypodense or isodense on non-contrast CT with either no significant enhancement or arterial enhancement	T1W: Hyperintense T2W: Iso- or hypointense and hyperintese rim. Suppression on opposed phase imaging

*Source*: Chiorean L, Cui XW, Tannapfel A, et al. Benign liver tumors in pediatric patients – Review with emphasis on imaging features. World J Gastroenterol. 2015;21(28):8541–8561. https://doi.org/10.3748/wjg.v21.i28.8541 and Chung EM, Cube R, Lewis RB, Conran RM. From the archives of the AFIP: Pediatric liver masses: Radiologic-pathologic correlation part 1. Benign tumors. Radiographics. 2010;30(3):801–826. https://doi.org/10.1148/rg.303095173

T1W, T1-weighted; T2W, T2-weighted; Y, yes; N, no; USG, ultrasoun; CEUSG, contrast enhanced ultrasound; NCCT, non-contrast CT scan.

## Hepatic haemangioma

These are the most common benign tumours in infancy and are seen in up to 10% of the paediatric population. They are similar to infantile haemangiomas of the skin and other organs which are the most common neoplasms of infancy.^4^

It is more common in preterm female children (female : male = 3:1)^6^ with no racial predilection. One-third are diagnosed in the first 3 months of life, while nearly 90% are diagnosed in the first 6 months. These patients present with an abdominal lump with no clinical symptoms. Although hepatic haemangiomas (HH) are benign, complications like rupture or haemorrhage can occur, which require necessary medical intervention. Depending on the age of presentation, the number of lesions and immunostaining for glucose transporter (GLUT-1), they are classified into congenital or infantile and focal, multifocal or diffuse. Glucose transporter-1 is the marker of skin lesions and infantile haemangiomas which are multifocal. Focal congenital haemangiomas are not associated with any skin lesions.

Percutaneous biopsy of a hepatic haemangioma carries an increased risk of haemorrhage. Liver biopsy is therefore contraindicated when a haemangioma is high in the differential diagnosis of a hepatic mass.

### Congenital hepatic haemangioma

Congenital lesions are usually focal lesions that are completely formed at birth and are detected antenatally during routine antenatal scans, and demonstrate involution after birth. Unlike the infantile type, congenital hepatic haemangiomas (CHHs) are GLUT-1-negative lesions. Congenital hepatic haemangioma mimics infantile hepatic haemangioma (IHH) on imaging, and hence the clinical as well as histopathological features are important distinguishing features. However, a large single lesion, heterogeneity, intravascular thrombi, ill-defined margin, large vascular spaces, calcification and necrosis favour CHH rather than IHH. On the contrary, IHHs are small, multifocal, iso- to hyperechoic lesions without areas of necrosis. Most CHHs show complete involution similar to their cutaneous counterparts and they are termed as rapidly involuting congenital haemangioma (RICH). Occasionally they can be partially involuting (PICH) or rarely noninvoluting (NICH).^3^

Antenatal ultrasound reveals a mixed, solid and cystic hepatic mass with increased blood flow on colour Doppler imaging. Foetal MRI shows an isolated vascular hepatic tumour appearing hypointense on T1-weighted (T1WI) and heterogeneously hyperintense on T2-weighted (T2WI). Foetal MRI can also reveal smaller lesions not detected on prenatal ultrasound.

**FIGURE 2:**
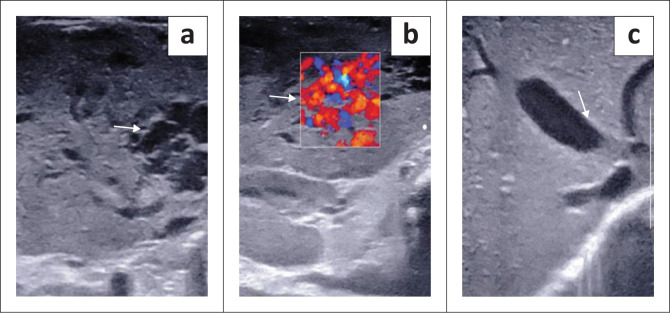
A 4-month-old female with congenital haemangioma diagnosed antenatally. Transverse greyscale ultrasound (a) with colour Doppler of the liver (b) shows an ill-defined iso- to hypoechoic lesion (arrow in a) with cystic spaces corresponding to vascular channels on colour Doppler in the left lobe of liver (arrow in b) and an ectatic middle hepatic vein (arrow in c).

Postnatal ultrasonography with colour Doppler plays a significant role not only in the diagnosis but also in follow-up. On greyscale, these lesions are usually single, large, hypoechoic lesions with internal vascular spaces, or they may sometimes demonstrate mixed echogenicity due to the presence of fibrosis, calcification, haemorrhage and necrosis, in contrast to IHHs and adult haemangioma.^7^ Doppler may show increased vascularity and enlargement of the hepatic artery, hepatic veins and inferior vena cava (IVC) due to portosystemic shunts. The lesion itself may show arterial and venous flow (Figure 2a–c). Imaging features suggestive of resolution are diminution of the velocities in the arteries, resolution of the arteriovenous shunt and decrease in the size of the lesions.^8^ Dilatation of the coeliac axis, hepatic artery and abrupt change of the abdominal aortic calibre below the origin of the coeliac axis are other typical features for this entity.^9^

**FIGURE 3:**
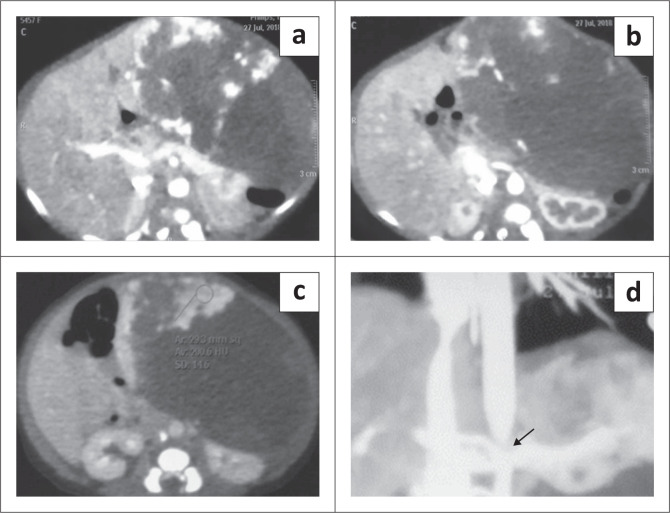
A 10-day-old female with a congenital hepatic haemangioma presented with left-sided abdominal swelling. Multiphase contrast-enhanced CT abdomen revealed a large, necrotic, exophytic mass lesion in the left lobe of the liver, almost completely replacing it. The lesion shows peripheral nodular enhancement in the arterial phase (a and b) with a large non-enhancing area. There is centripetal enhancement; however, the central portion remains hypodense even on the delayed phase (c). Note the attenuation of the abdominal aorta distal to the origin of the coeliac axis on the coronal maximum intensity projection image (arrow in d).

On CT, these lesions are large and show intense arterial phase enhancement and may show areas of calcification and necrosis. Non-enhancement of the necrotic component is seen even on delayed imaging (Figure 3a–d).

These lesions are hypointense on T1WI and hyperintense on T2WI MR images and show rapid arterial enhancement with areas of necrosis and calcification.^10^ Congenital haemangiomas are rarely associated with cutaneous haemangiomas.^11^

**FIGURE 4:**
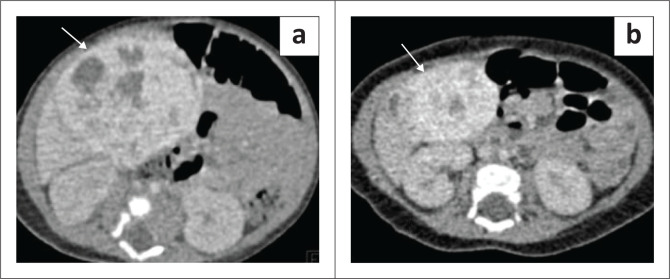
Congenital hepatic haemangioma in a 3-month-old female. Axial contrast-enhanced CT (CECT) through the abdomen shows a large exophytic heterogeneous lesion in the right lobe of liver (arrow in a). Follow-up CECT after 3 months showed significant spontaneous decrease in the size of the mass (b).

Most of the congenital haemangiomas resolve spontaneously (Figure 4); however, non-revolving lesions may require medical treatment with propranolol or steroids. Embolisation can be performed for cases not responsive to medical therapy.^11^

Differentials include mesenchymal hamartoma (MH), hepatoblastoma and neuroblastoma metastasis. Various imaging features and biochemical markers can be helpful in excluding other differentials. Mesenchymal hamartoma reveals a multiseptated cystic appearance and alpha-foetoprotein (AFP) levels are elevated in hepatoblastoma.

### Infantile or multifocal haemangioma

Infantile hepatic haemangioma can be solitary or multifocal and they are similar to their cutaneous counterparts, demonstrating rapid proliferation, followed by involution. They are GLUT-1-positive lesions. Most patients are asymptomatic, but rarely present with congestive heart failure due to arteriovenous shunting. Concurrent cutaneous lesions are seen occasionally.

**FIGURE 5:**
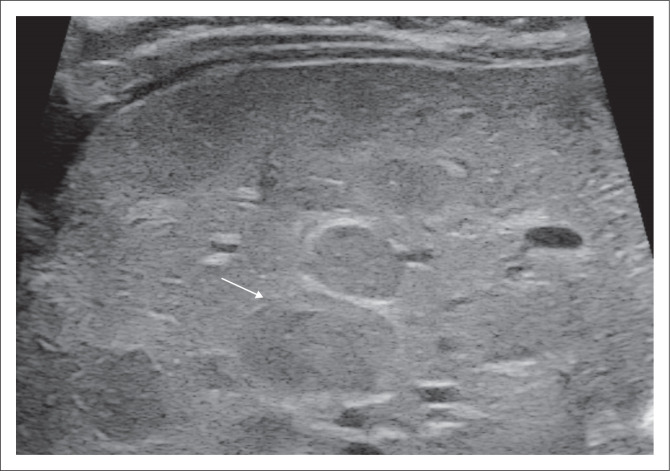
Infantile hepatic haemangioma in an 8-month-old child. Transverse grayscale ultrasound showing multiple well-defined isoechoic lesions in both the lobes of liver.

Ultrasound and colour Doppler reveal multiple, hyperechoic or isoechoic lesions with or without prominent vascular spaces (Figure 5). Lesion vascularity is variable.^4^ Contrast-enhanced ultrasound reveals the classic pattern of discontinuous peripheral nodular enhancement on the arterial phase and gradual centripetal fill-in on the portal venous phase in most cases.^4^

**FIGURE 6:**
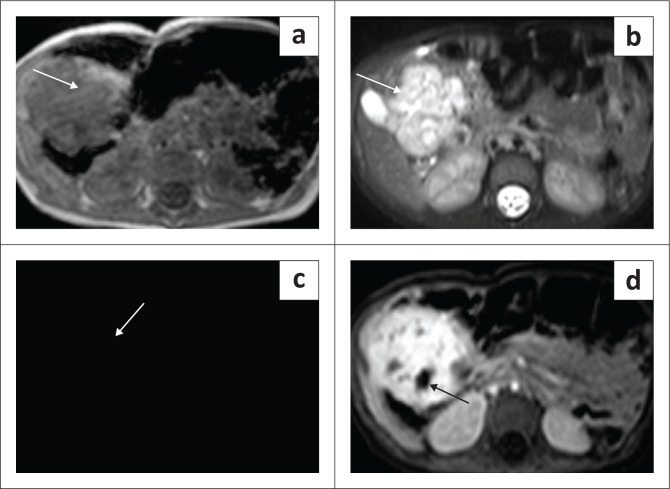
Axial T1-weighted (T1W) image (a) and axial T2-weighted (T2W) image (b) showing a well-marginated lesion, brightly T2 hyperintense, in the right lobe of liver. Dynamic contrast-enhanced axial T1W fat-saturated images (c and d) showing peripheral nodular enhancement in the arterial phase (arrow in c) with centripetal filling and almost complete enhancement with a few nonenhancing areas (arrow in d). The above features are typical for a solitary infantile hepatic haemangioma.

**FIGURE 7:**
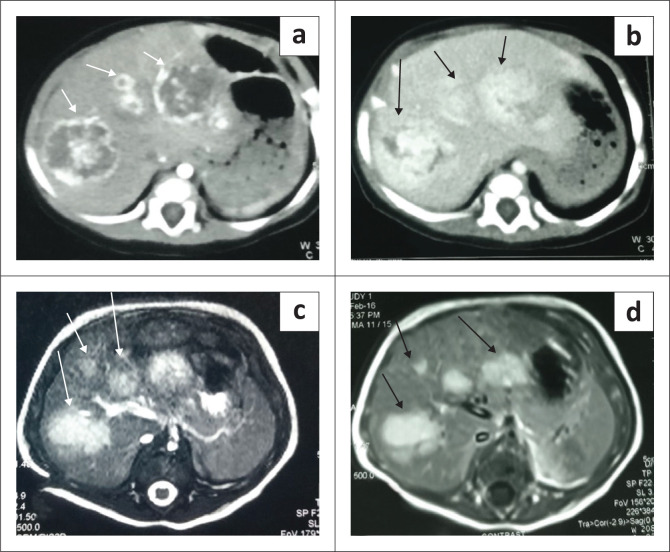
A 2-month-old female with multifocal infantile haemangioma. Multiphase CT (a and b) and MRI (c and d) through the liver show multiple focal lesions in both the lobes of the liver with peripheral nodular enhancement on the arterial phase (arrows in a) and progressive centripetal enhancement with near-complete filling on the delayed phase (arrow in b). Axial T2-weighted (T2W) image shows multiple hyperintense lesions in both the lobes of liver (arrows in c) and delayed phase post-contrast axial T1 MRI of the same patient shows near-complete filling similar to the CT scan (arrows in d).

The CT and MRI appearances of IHHs are similar to adult hepatic haemangiomas with peripheral nodular enhancement, centripetal filling and complete uniform enhancement on the delayed phase (Figure 6 and Figure 7). Multifocal lesions are associated with congestive cardiac failure due to arteriovenous and portovenous shunting. Infantile hepatic haemangiomas are associated with cutaneous haemangiomas in 60% of cases.^12^

Most IHHs show spontaneous resolution and a good response to propranolol, which is currently the preferred treatment. Earlier, corticosteroids and interferon-alpha were used for treating non-resolving cases. However, side effects like spastic diplegia were observed with interferons and a 20% – 40% failure rate was seen with corticosteroids, in addition to the significant side effects of growth retardation, cushingoid syndrome and obesity. Embolisation can be performed in cases non-responsive to medical therapy.^11^

### Diffuse haemangioma

Diffuse lesions are GLUT-negative lesions and show complete replacement of the hepatic parenchyma. Arteriovenous, arterioportal and portovenous shunting can be seen which can lead to high-output cardiac failure. Complications such as abdominal compartment syndrome and hypothyroidism due to overproduction of type III iodothyronine deiodinase have been described.^13^

Management requires monitoring of cardiac function and T3, T4 and TSH levels. Resolution of thyroid hormone abnormalities occurs with the involution of lesions; hence their levels are an important marker for the resolution or progression of lesions. Non-resolving or complicated cases require embolisation or surgical resection.

Most hepatic haemangiomas are non-progressing and do not require treatment. In a small number of cases, rapid volumetric growth or complications on follow-up prompt further appropriate therapy.^11^

## Mesenchymal hamartoma

Mesenchymal hamartoma is the second most common benign tumour after IHH and is usually diagnosed before the age of 2 years, latest by 5 years. It affects males more than females (male : female ratio 3:2).^14^ It can also be diagnosed in utero and can be associated with hydrops fetalis.^15^ The majority of lesions are asymptomatic; however, they can present with an abdominal mass and may manifest with mass effect related to rapid enlargement from fluid accumulation within the cysts.^16^

Biochemical markers, including AFP, are usually unremarkable but can sometimes be elevated and MH can therefore be confused with hepatoblastoma. Elevated AFP in MH is likely due to hepatocytes and bile duct epithelium lying in a loose myxoid stroma. Few of them present with non-specific clinical features such as abdominal pain, anorexia, diarrhoea or weight loss.^15^ Inferior vena cava compression can be seen when the mass is large and can lead to lower limb oedema. It can also present with respiratory distress due to elevation of the diaphragm. Large lesions may rupture necessitating emergency intervention.^15^

In utero, MH is diagnosed in the third trimester as a solid or complex, multicystic, well-circumscribed lesion just above the kidney on ultrasound. The organ of origin may not be apparent in all cases.^15^

**FIGURE 8:**
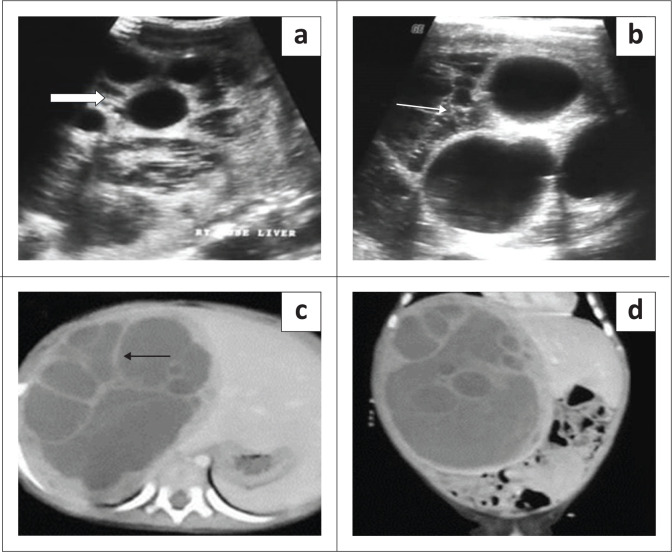
Mesenchymal hamartoma in a 9-month-old child. Transverse greyscale ultrasound (a) and (b) shows a multiseptated, cystic mass in the right lobe of liver (arrow in a) with no vascularity. Axial and coronal reformatted contrast-enhanced CT images of the upper abdomen in a different child show a large, peripherally enhancing, multiseptated, cystic lesion in the right lobe of liver with hepatomegaly. Enhancement of the septae (arrow in c) is also seen.

**FIGURE 9:**
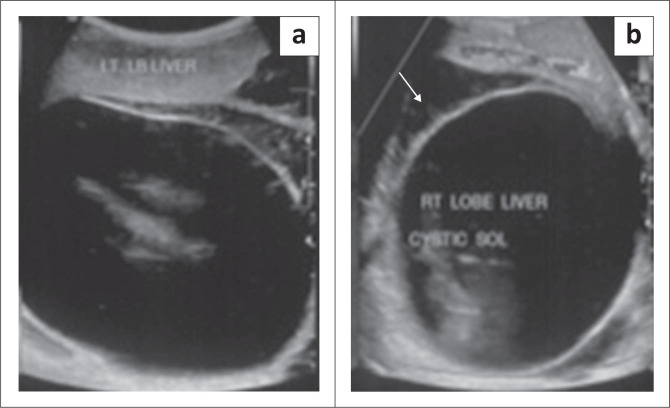
A 1-year-old female with a mesenchymal hamartoma presented with an abdominal mass. Transverse greyscale ultrasound abdomen (a and b) revealed a large, unilocular, cystic mass in the left lobe of liver with adjacent fluid (arrow in b) suggestive of localised rupture.

**FIGURE 10:**
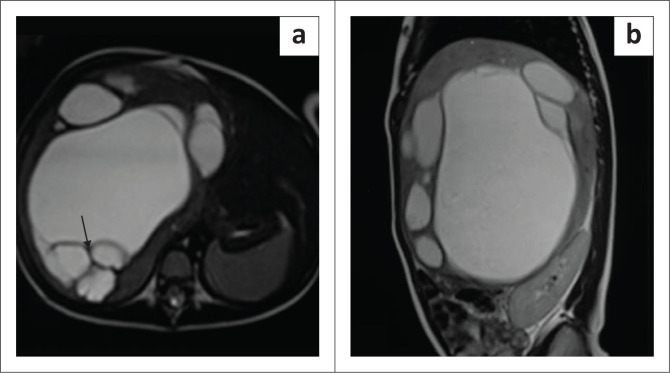
Mesenchymal hamartoma in a 2-year-old female. Axial and sagittal T2-weighted (T2W) MRI images (a and b) of the abdomen showing a multiseptated, hyperintense lesion occupying the right lobe of liver with internal hypointense septae (arrow in a). The lesion is indistinguishable from a hydatid cyst on imaging.

On postnatal ultrasound in infants and young children, the mass is predominantly cystic or can have a mixed, cystic-solid appearance depending upon the predominant component (mesenchymal and stromal). Rarely, it can present as a purely solid lesion. The cystic component of the tumour can be purely anechoic with echogenic septations (Figure 8a–b) or can show internal debris or fluid-debris levels depending on the content (Figure 9a–b). Due to the lack of a tumour capsule, the tumour can grow to a very large size.^4^ Loculated fluid adjacent to the lesion in the subcapsular or perihepatic location suggests rupture (Figure 10a–b). Colour Doppler interrogation shows minimal vascularity. Ultrasound-guided intraoperative aspiration of fluid from the cystic components of the tumour to reduce its volume can facilitate surgical resection.^15^

Imaging with NCCT may show a multiseptated cystic or solid-cystic lesion. The cystic component shows fluid attenuation. Septations and solid components show enhancement on post-contrast studies (Figure 8c–d).^5^

On MRI, the cystic components are hypointense on T1WI and demonstrate high, intermediate or low signal on T2W1 depending on the proteinaceous content. Septations and walls are hypointense on both T1W1 and T2W1 images (Figure 10a–b) and display post-contrast enhancement as on CT. The solid variant is hypointense on both T1WI and T2WI and shows homogeneous contrast enhancement.^17^

Benign differentials of predominantly cystic MH variants include simple cyst, hydatid cyst and abscess if the mass is entirely intrahepatic, and choledochal cyst, duplication cyst and mesenteric lymphangioma if the mass is partly extrahepatic.^5^ Mesenchymal hamartoma can closely resemble hydatid cyst; however, the age of presentation and serology can help in their differentiation.^18^

Undifferentiated embryonal sarcoma (UES) is a malignant tumour that can also closely mimic the cystic variant of MH on CT and MRI; however, older age of presentation (6–10 years), as well as a solid appearance on ultrasound, can be used to differentiate UES from MH. Some consider MH as the precursor for UES. Solid variants can resemble hepatoblastoma; however, the presence of calcification in around 50% and raised AFP in hepatoblastoma can be used as differentiating features.^19^

Resection is the treatment of choice, and in non-resectable cases, enucleation and marsupialisation are performed.^10^

## Focal nodular hyperplasia

These are rare tumours in children and constitute only 2% – 7% of all paediatric liver tumours.^20^ They are more common in adult females between the ages of 35 and 50 years. In children, they can occur at any age with a mean age of 7 years. The underlying pathogenesis is not known with a postulated theory that FNH is the result of an underlying vascular malformation such as vascular dysplasia, Budd-Chiari syndrome or vasculitis. Most patients are asymptomatic; however, it can present with non-specific abdominal pain or a mass.^5^ The incidence of FNH increases in children with a prior history of radiotherapy or chemotherapy. It was found that multiple FNHs in children without a previous history of malignancy were larger and had central scars, while those with a previous history of malignancy were of small size without the central scar.^21,22^

**FIGURE 11:**
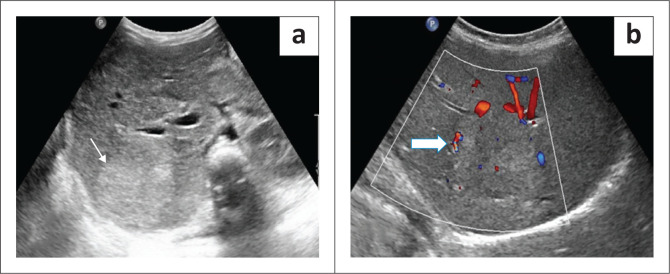
Focal nodular hyperplasia in a 5-year-old female who presented with abdominal pain. Transverse greyscale ultrasound (a) and colour Doppler (b) showing a well-circumscribed hyperechoic mass in the right lobe of the liver (thin arrow in a). The mass is showing mild vascularity on colour Doppler (thick white arrow).

On ultrasound, FNH is typically a well-circumscribed mass with variable echogenicity and a central hyperechoic scar (seen in approximately 50%). Scar calcification is very rare.^23,24^ Doppler can show the spoke wheel type of vascularity with increased flow in the central scar, extending to the periphery (Figure 11).

Arterial phase contrast-enhanced ultrasound shows centrifugal hyperenhancement or spoke wheel enhancement^23,24^ and portal venous phase shows an isoenhancing lesion with no washout.^4^

**FIGURE 12:**
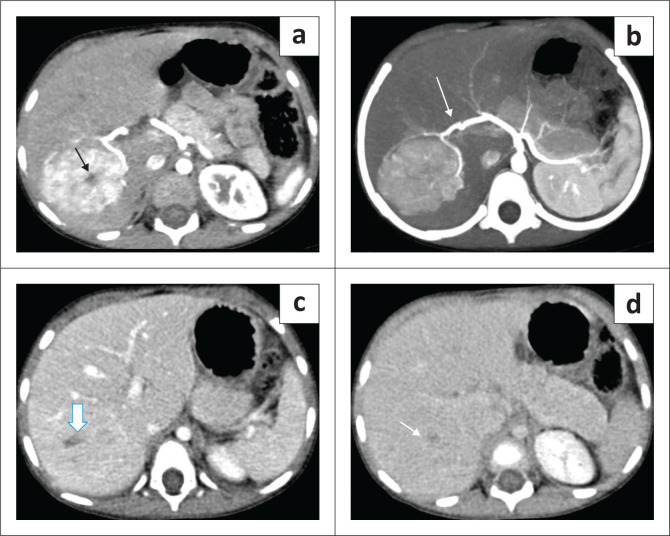
Focal nodular hyperplasia in a 5-year-old child. Dynamic contrast-enhanced CT scan of the abdomen showing an arterially enhancing mass in the right lobe of the liver with a hypodense central scar (black arrow in a). Maximum intensity projection image shows arterial feeders from the right hepatic artery (arrow in b). Persistent enhancement is seen in the lesion on the portal venous (c) and the delayed phase (d) shows some enhancement of the central scar.

Typical features of FNH on CT are a round hypo- to isodense mass on NCCT that demonstrates homogeneous enhancement in the arterial and early portal venous phase (more than the liver parenchyma) and appear isodense to the adjacent liver parenchyma on the delayed phase. The central scar is usually hypoenhancing in the arterial phase and shows enhancement on delayed phase images. The central scar does not show calcification (differentiating it from fibrolamellar carcinoma [FLC]) (Figure 12). Atypical features like peripheral enhancement, washout in portal venous phase or lack of enhancement of the central scar on delayed phases scan warrant biopsy of the lesion.^25,26^

**FIGURE 13:**
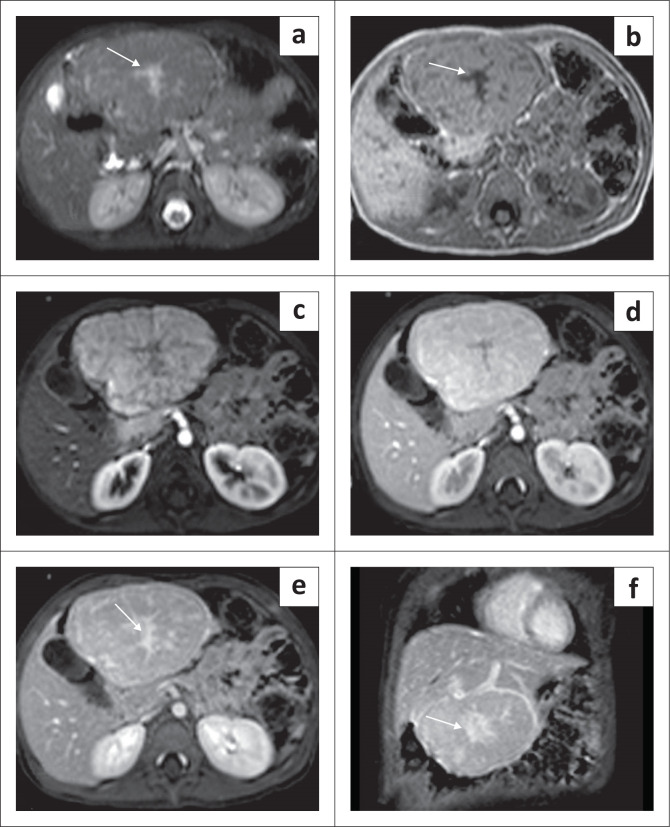
MRI of a 3-year-old female with focal nodular hyperplasia. T2-weighted (T2W) fat saturated axial image of the abdomen showing a well-circumscribed isointense lesion with a hyperintense central scar (arrow in a) in the left lobe of the liver. The lesion is isointense on the T1-weighted (T1W) sequence (b) and the central scar is hypointense (arrow in b). The lesion is hypervascular on the arterial phase (c) and shows contrast retention on portal venous (d) and delayed phases (e). The central scar is hypointense on both the arterial and portal venous phases. Delayed scans axial (e) and coronal (f) image show enhancement of the central scar (arrow in e and f).

On MRI, FNH is typically hypo- or isointense on T1WI and hyper to isointense on T2WI. It has been referred to as the ‘stealth lesion’ as it closely matches the signal intensity of the background liver making it difficult to detect. They show hyperenhancement on the arterial and portal venous phases. The central scar is hypointense on T1WI and hyperintense on T2WI due to the myxoid content and shows enhancement on the delayed phase which is a differentiating feature from FLC (Figure 13a–f). The scar in FLC is hypointense on both T1WI and T2WI due to its collagen content and does not show enhancement in the delayed phase.^27^ Additionally, as FNH contains normal hepatocytes with functioning organic anion transporting polypeptide (OATP), it takes up hepatobiliary contrast agents such as gadobenate dimeglumine and gadolinium EOB-DTPA. However, due to malformed biliary ducts, the contrast is retained within the lesion and the lesions therefore appear hyperintense on the hepatobiliary phase, a feature which can be used to differentiate FNH from adenoma and metastasis.^26^ Hepatic adenoma (HA) is hypointense while FNH is hyperintense on the delayed phase.^26^

## Hepatic adenoma

Hepatic adenomas (HAs) account for 2% – 4% of all paediatric liver tumours.^28^ Depending on the genetic and pathologic subtypes, HAs are classified into four subtypes^4^:

- Inflammatory adenoma.
- Hepatocyte nuclear factor 1 alpha (HNF-1ɑ) mutated hepatocellular adenoma.
- β-Catenin mutated hepatocellular adenoma.
- Unclassified.

They are solitary in about 70% – 80% of cases.^4^ Multiple adenomas are more frequently encountered in children than in adults, and more than 10 adenomas without underlying glycogen storage disease or steroid use are termed multiple adenomatosis.^29^ Predisposing factors such as glycogen storage disease, anabolic steroid treatment, Hurler syndrome, Turcot syndrome, immunodeficiency syndrome, tyrosinaemia, galactosemia, diabetes mellitus and germline mutation of *HNF*-ɑ gene are seen in adenomatosis.^30,31^ Multiple lesions are associated with haemorrhage and a younger age of presentation is typically seen in children with glycogen storage disease and is commonly associated with the beta-catenin type and lack of HNF-ɑ mutation.^32^ The postulated mechanism for the development of adenomas in these groups of patients is related to hepatic vascular injury due to steroid therapy.^32^ They are mostly asymptomatic or occasionally present with abdominal pain.

On ultrasound, the lesions are usually large and heterogeneous with areas of hyperechogenicity due to fat and haemorrhage and may have cystic areas. Ultrasound is also performed annually as a screening tool in children with the predisposing conditions described earlier.^28^ Change in size and shape should raise the suspicion of malignancy.^33^ The lesions are vascular and show either an arterial or venous waveform on Doppler ultrasound. In contrast to FNH in which there is central arterial flow, HAs show homogeneous arterial flow. Arterial phase CEUS shows a hypervascular lesion relative to the adjacent liver and an isoechoic lesion on portal venous phase with partial washout due to the absence of portal veins. Delayed washout is seen in HNF-1α and inflammatory adenoma.^4^

In contrast to FNH, adenomas are typically heterogeneous masses on CT due to the presence of calcification (seen in 5% – 15% of cases) or areas of fat (7% – 10% of cases).^33^ Haemorrhage within the tumour can be seen as hyperdensity on NCCT. Haemorrhage is most common in the inflammatory subtype. These lesions show enhancement in the arterial phase, retain contrast in the portal venous phase and may show washout in the delayed phase. The absence of a scar and the heterogeneous appearance of HAs can be used to differentiate them from FNH.^34^

**FIGURE 14:**
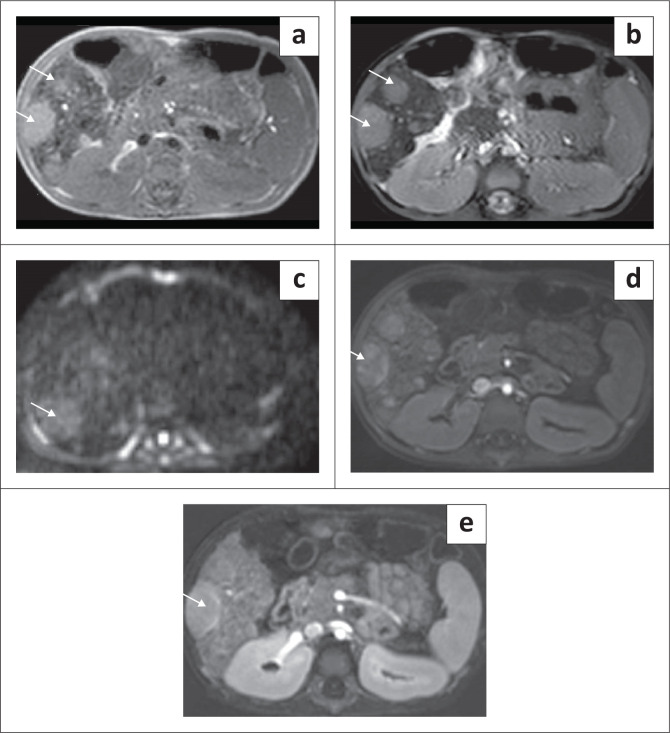
MRI of multiple liver adenomas in a child with glycogen storage disorder. Axial T1-weighted (T1W) (a) and T2-weighted (T2W) fat saturated (b) images of the upper abdomen of a 7-year-old child showing a few hyperintense lesions (arrows) in the right lobe of liver. The background liver intensity is abnormal, and the liver has nodular outline. Diffusion weighted trace image (c) showing mild restricted diffusion in the lesion (apparent diffusion coefficient [ADC] not shown). Dynamic contrast-enhanced images showing arterial enhancement (d) of the lesions (arrow) with retention of contrast in the portal venous phase (e).

On MRI, HAs are usually heterogeneous on T1 and T2W images. The HNF-ɑ subtype, due to the presence of intracellular fat, can appear hyperintense on both T1W and T2W images with signal loss on chemical shift imaging.^35^ Post-contrast enhancement on MRI is very similar to that of CECT (Figure 14). Due to the presence of fat and arterial enhancement, these lesions have to be differentiated from fat-containing HCC. The presence of a pseudocapsule on the delayed phase in HCC can be used as a differentiating feature.^33^ Unlike FNH, HA shows hypointense signal on the hepatobiliary phase obtained after hepatocyte-specific contrast agents.^33^

Hepatic adenomas are prone to various complications including rupture, haemorrhage and malignant transformation. Hepatic adenomas, which are single, large (> 5 cm), associated with glycogen storage disease or male gender, are usually resected. In cases of multiple lesions, the largest lesion can be resected and the rest can be followed up. Smaller lesions (< 5 cm) are either followed up or resected depending on other associated risk factors.^33^ Surgical resection can also be done whenever there is suspicion of malignant transformation into HCC. Haemorrhage is seen in approximately 10% of patients predominantly affecting the larger lesions. Bleeding can be contained within the tumour or may extend into the subcapsular region or peritoneal cavity in which case a child can present acutely with sudden abdominal pain or hypovolemic shock. Malignant transformation is rare in children and is reported in children with glycogen storage disease, Fanconi anaemia and those on steroid therapy.^36^

## Nodular regenerative hyperplasia

This is also a rare entity in the paediatric age group and is seen in only 4.5% of children.^37^ Underlying pathogenesis for the development of these lesions is the microvascular disturbances related to intrahepatic venous or arterial vessels or any changes in the sinusoidal walls causing vascular obliteration or thrombus which would subsequently cause repeated atrophy and compensatory regeneration of the liver.^4^ Although most of these cases occur in association with various systemic diseases like myeloproliferative syndrome, thrombocytopaenia, pancytopaenia, collagen vascular disease, and Budd-Chiari syndrome or due to cytotoxic or immunosuppressive drugs, some cases may also be idiopathic.^38^ Incidence of nodular regenerative hyperplasia (NRH) is relatively higher after a liver transplant and can be seen in around 1% of those patients.^39^

Biopsy is the confirmatory test that can show multiple regenerative micronodules without parietal thickening of the portal venules or scar. Fibrosis is absent.^4^

Multiple iso- to hypoechoic well-circumscribed nodules are seen on ultrasound, usually measuring less than 5 mm. However, when the lesions are too small and multiple, the liver can appear heterogeneous in echotexture on ultrasound. Rarely, these lesions are hyperechoic with a lucent centre and can mimic metastasis.^40^

On CT, NRH nodules show varied imaging features as described in the published literature. These nodules may appear hypo- or isodense on NCCT with no significant enhancement or they may show arterial phase enhancement. They appear either hypo- or isodense on the portal venous phase.^41,42^

**FIGURE 15:**
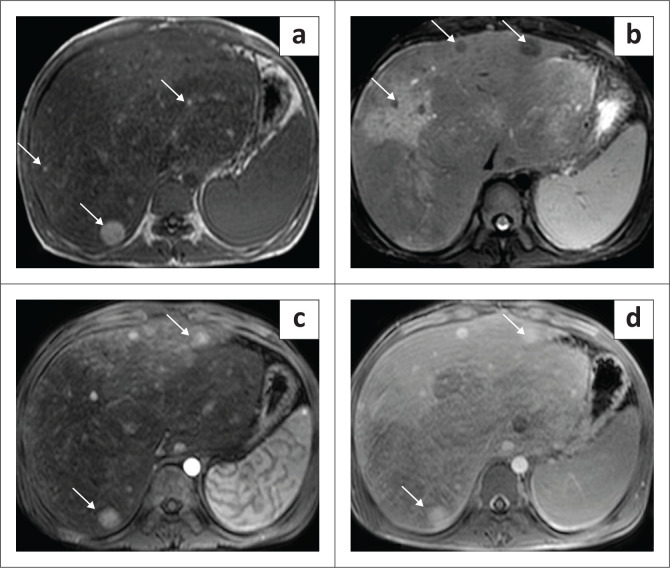
(a–d) MRI of multiple nodular regenerative hyperplasia in a child with hepatic venous outflow obstruction. Axial T1-weighted (T1W) (a) and T2-weighted (T2W) fat saturated (b) images of the upper abdomen showing multiple tiny lesions which are hyperintense and hypointense, respectively, involving both the lobes of liver. There is associated hepatomegaly. The post-contrast arterial phase image (c) shows intense contrast enhancement of all the lesions (arrows) which also demonstrate contrast retention on the portal venous phase (d).

Due to fat content, these lesions appear hyperintense on T1WI and iso- to hypointense with a hyperintense rim on T2W2. They may show suppression of signal on opposed phase imaging. Post-contrast images demonstrate features similar to what has been described on CT (Figure 15).^4,41,42^

**FIGURE 16:**
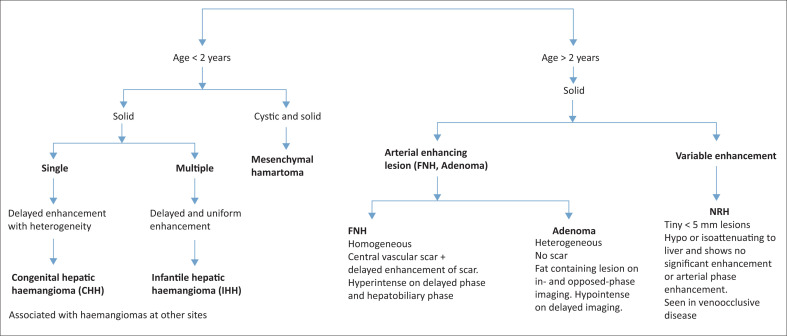
Diagnostic algorithm for benign liver lesions in the paediatric age group.

An algorithm on how to approach and reach an appropriate diagnosis in benign liver lesions in the paediatric age group is presented in Figure 16.

## Conclusion

Benign liver lesions are not rare in children and need to be carefully evaluated on imaging. Infantile hepatic haemangioma and MH are two benign lesions that are exclusively seen in children. Hepatic adenomas are frequently multiple in children due to the underlying predisposing conditions. Most benign lesions are associated with a normal AFP; however, it is important to know that high levels can be normal in early infancy and tend to normalise after 6 months of life. It is also important to be aware of the complications so that they are managed appropriately.
